# Effects of Crocodile Oil (*Crocodylus siamensis*) on Liver Enzymes: Cytochrome P450 and Glutathione S-Transferase Activities in High-fat DietFed Rats

**DOI:** 10.1155/2022/9990231

**Published:** 2022-11-22

**Authors:** Pitchaya Santativongchai, Krittika Srisuksai, Kongphop Parunyakul, Piriyaporn Thiendedsakul, Preeda Lertwatcharasarakul, Wirasak Fungfuang, Phitsanu Tulayakul

**Affiliations:** ^1^Bio-Veterinary Sciences (International Program), Faculty of Veterinary Medicine, Kasetsart University, Bangkok 10900, Thailand; ^2^Department of Zoology, Faculty of Science, Kasetsart University, Bangkok 10900, Thailand; ^3^Animal Health and Biomedical Science, Faculty of Veterinary Medicine, Kasetsart University, Bangkok 10900, Thailand; ^4^Department of Pathology, Faculty of Veterinary Medicine, Kasetsart University, Kamphaeng Saen Campus, Nakhon Pathom 73140, Thailand; ^5^Department of Veterinary Public Health, Faculty of Veterinary Medicine, Kasetsart University, Kamphaeng Saen Campus, Nakhon Pathom 73140, Thailand; ^6^Kasetsart University Research and Development Institute (KURDI), Kasetsart University, Bangkok 10900, Thailand

## Abstract

Crocodile oil is a highly effective treatment for ailments ranging from skin conditions to cancer. However, the effects of the oil on liver detoxification pathways are not well studied. This study aimed to investigate the effects of crocodile oil on the detoxification enzyme activities and the mRNA expressions of cytochrome P450 1A2 (CYP1A2), cytochrome P450 2E1 (CYP2E1), and glutathione S-transferase (GST) in rats. The rats were divided into four groups (*n* = 7/group): rats received a standard diet (C), a high-fat diet or HFD (H), and HFD with 1 ml (HCO1) and 3 ml (HCO3) of the oil per kg body weight. Interestingly, the oil yields from this study presented alpha-linolenic acid (0.96%) at similar levels compared with fish oil. The results revealed that HFD significantly increased the activity and relative gene expression of CYP1A2 in the H group (*P* < 0.05), whereas 3% crocodile oil normalized the enzyme activities compared to the C group. This suggested inhibiting the HFD-induced expression of CYP1A2 mediated by the omega-3 fatty acids found in the oil. Also, crocodile oil supplementation did not reduce the activities of GST. However, the relative gene expression of GSTA1 was significantly decreased (*P* < 0.05) in the HCO1 and HCO3 groups compared to the H group, which might be attributed to the lower lipid peroxidation that occurred in the liver tissues. Therefore, it could be suggested that using crocodile oil could help in liver detoxification through the CYP1A2 even when offered with a HFD.

## 1. Introduction

Obesity is caused by an imbalance between energy intake and expenditure. This condition has become a global epidemic, with over 650 million affected adults [1]. Obesity is associated with many chronic diseases such as cardiovascular diseases, type 2 diabetes mellitus (T2DM), metabolic syndromes, and fatty liver disease [2–4]. A high-fat diet (HFD) induces overconsumption and weight gain, leading to obesity [5]. Moreover, HFD and a low-carbohydrate diet revealed both intracellular and extracellular adaptations that have been shown to elicit favorable cardio-metabolic changes associated with obesity [6].

Crocodile oil (CO) is one of the traditionally used natural oils documented to be highly effective in treating several ailments ranging from skin conditions to cancer. For example, the oil is used to alleviate illnesses including asthma, influenza, and a constant phlegmatic cough [7]. Moreover, a previous study found that the oil had antimicrobial activities against *Staphylococcus aureus* and *Klebsiella pneumonia*, and it also exhibited fungal specificity against *Candida albicans*. Also, the anti-inflammatory response results showed that the oil exhibited a relatively short duration of action from the oral administration route and an acute, relatively long action from the topical application [8]. Additionally, the oil from *Crocodylus siamensis* could heal burn wounds, create new skin cells, and collect skin collagen [9]. However, the effects of the oil on liver detoxification pathways have not been investigated.

The liver reacts to drugs and toxins once they enter the bloodstream. Many of these toxic compounds are fat soluble; thus, they are difficult to excrete from the body. Accordingly, the role of liver detoxification pathways is to convert these compounds into less harmful and more soluble forms, which are readily excreted from the body [10]. Liver detoxification typically involves two sets of chemical pathways. Phase I detoxification is the first part of the defense mechanism. Toxins are converted into intermediate forms by oxidation, reduction, and hydrolysis reactions [11, 12]. These reactions are mediated by a group of enzymes known as the cytochrome P450 superfamily of enzymes (CYP450). However, these conversions produce damaging free radicals, which can still pose a toxic threat to the body [10, 12]. Therefore, it is the role of phase II detoxification to provide final neutralization of the byproducts and other remaining toxins. The converted chemicals are then attached to another substance via a conjugation reaction [13, 14]. This renders the compounds even less harmful and makes them water-soluble. The water-soluble compounds can then be excreted from the body through urine via the kidneys. One of the critical phase II detoxification enzymes is a group of glutathione S-transferases (GSTs), which are found mainly in the cytosol [14, 15].

CYP450 is generally the first defense employed by the body to biotransform various xenobiotics, steroid hormones, and pharmaceuticals [12]. The CYP1A family participates in the metabolism of procarcinogens, hormones, and pharmaceuticals [16]. The major hepatic isoform of the CYP1A family is CYP1A2 [17]. It is well known for its role in the carcinogenic bio-activation of polycyclic aromatic hydrocarbons (PAHs), heterocyclic aromatic amines or amides, polychlorinated biphenyls (PCBs), and other endogenous and exogenous substances [16, 18]. At the same time, CYP2E1 is well known for metabolizing nervous system agents such as halothane, isoflurane, chlorzoxazone, and ethanol. Moreover, it also bio-activates procarcinogenic nitrosamines and aflatoxin B1, a hepatocarcinogen that causes liver cancer [11]. Another targeted enzyme in this study is glutathione S-transferase (GST), whose primary function is to catalyze the conjugation of glutathione (GSH) conjugates for the biotransformation of metabolites [14]. Additionally, it is widely recognized that antioxidant enzymes, such as superoxide dismutase (SOD), catalase (CAT), and GST, exert natural and essential defenses against oxidative impairment [19]. Consequently, this study focused on these three kinds of detoxification enzymes to determine the detoxification effects of oral administration of CO.

Therefore, this study aimed to investigate the effects of CO supplementation on the liver detoxification enzyme activities of CYP1A2, CYP2E1, and GST and the gene expressions in rats that were fed a HFD The insight gained from this study could be applied to further investigate the hepatoprotective mechanism induced by CO.

## 2. Materials and Methods

### 2.1. Oil Extraction

Oil samples were obtained from the abdominal fat of Siamese crocodile (*Crocodylus siamensis*) certified by Good Farm Practice (GFP) crocodile farms in Nakhon Pathom and Kanchanaburi, Thailand. The samples were then stored at −20°C until the extractions were performed.

According to a previous study, CO was extracted using the wet cold-pressed method [20]. In brief, the fat samples were mechanically pressed through two layers of filter cloth with distilled water using a proportion of 1 : 1 (w/v). The solution was subsequently stored until the transparent oil layer was separated. The oil layer was then evaporated and stored at 25°C until used.

### 2.2. Fatty Acid Composition

The identification and quantification of the fatty acids were conducted by gas chromatography (Agilent 7890B, Santa Clara, CA, USA). The instrument was fitted with a flame ionization detector, and fatty acid separation was conducted on a fatty acid methyl ester column (CP-Sil 88, Agilent, Santa Clara, CA, USA) with a length of one hundred meters, an internal diameter of 0.25 mm, and a stationary phase film thickness of 0.20 *µ*m. Identification of fatty acids was achieved by comparison of the retention times with authentic standard fatty acid methyl esters.

### 2.3. Animal Treatments

Male Wistar rats (24 weeks old) were obtained from the Nomura Siam International Co., Ltd., M-CLEA Bioresource Co., Ltd., Bangkok, Thailand.

The rats were housed in a temperature-controlled room (25 ± 2°C) at 60–70% humidity and a 12 h artificial light/dark cycle. The rats were randomly divided into four groups (7 rats/group): rats received standard diet (C, control group), HFD (H), HFD supplemented with 1 ml of CO (HCO1) per kg body weight, and HFD supplemented with 3 ml of CO (HCO3) per kg body weight. Rats in the same group were housed together. All groups were fed *ad libitum* with the standard diet (51.00% carbohydrates, 4.60% fats, and 24.90% proteins: Nomura Siam International Co., Ltd., Bangkok, Thailand) and HFD (27.46% carbohydrates, 41.04% fats, and 20.18% proteins) until the end of the experiment (Table 1). After 20 weeks of the experimental period, the HCO1 and HCO3 groups were supplemented with the extracted CO by oral gavage once daily for nine weeks, whereas the C and H groups received water instead.

### 2.4. Sample Collection

At the end of the experimental protocol, rats were fasted for 8–12 h and euthanized for liver dissection by intraperitoneal injection of 40 mg/kg pentobarbital sodium. The liver samples were dissected and flushed with a cold sucrose buffer (0.25 M sucrose, 1 mM EDTA, Tris-HCL 20 mM, pH 7.4) and stored at −80°C until analyses were performed.

### 2.5. Cytosolic and Microsomal Fractions

The cytosolic and microsomal fractions were extracted by using the method from the previous studies [21, 22] with slight modifications. In brief, 4 g of frozen liver tissues was homogenized in homogenization buffer (0.25 M sucrose, 0.2 mM DTE, 1 mM EDTA, 10 mM Tris-HCl, pH 7.4). The samples were then centrifuged at 10,000*g* for 10 min. Subsequently, the supernatant was ultra-centrifuged at 105,000*g* for 1 h. The supernatant, the cytosolic fraction, was stored at −80°C. After that, the pellet was re-homogenized in sucrose buffer and ultra-centrifuged again. The pellet, the microsomal fraction, was then stored at −80°C until analyzed. The protein concentrations were measured by the method of Bradford [23] by using a reagent for protein assay (Bio-Rad Protein Assay, Bradford Reagent catalog number B6916, Sigma-Aldrich, Inc., MO, USA), and bovine serum albumin (Albumin, Bovine Serum, Fraction V, RIA and ELISA Grade, Sigma-Aldrich, Inc., MO, USA) was used as the standard protein. The determination was achieved by using an ultraviolet (UV)-visible spectrophotometer (BioTek Synergy H1, Winooski, VT, USA) at 595 nm.

### 2.6. Enzyme Activities

Determination of CYP450 activity in the microsomal fraction was performed according to the method of a previous study [24]. In brief, the microsomal fractions (0.25 mg protein) were added to react with the reaction mixture containing 100 *µ*M 3-cyano-ethoxy-coumarin (CEC, a substrate for CYP1A2), 2.6 mM *β*-NADPH, 3 mM MgCl_2_, 0.8 U glucose-6-phosphate dehydrogenase (G-6-PD), 6.6 mM glucose-6-phosphate (G-6-P), and 50 mM potassium phosphate buffer pH 7.4 by using the excitation wavelength of 409 nm and an emission of 460 nm. Additionally, in the same way as CYP1A2, 7-methox-4-trifluoromethyl coumarin (7-MFC, a substrate for CYP2E1) was used instead at the excitation of 409 nm and the emission of 530 nm for detection of CYP2E1 activities. The enzyme activities were determined by using a fluorescent spectrophotometer (BioTek Synergy H1, Winooski, VT, USA).

GST activity was detected using BioTek Synergy H1 microplate reader (Winooski, VT, USA) according to the previous studies [25, 26] with slight modifications. In brief, the cytosolic fractions (1 mg protein) were added to start the reaction with 1 mM 2,4-dinitrochlorobenzene (CDNB, a substrate for universal-GST), 1 mM GSH, and 0.1 M sodium phosphate buffer (pH 6.5). The activity was then determined by using the UV-visible spectrophotometer at 340 nm.

### 2.7. Reverse Transcription-Quantitative Polymerase Chain Reaction (RT-qPCR)

The total RNA in each 30 mg liver tissue sample was extracted using an RNeasy® Mini Kit (Qiagen, Inc., Hilden, Germany). Removal of genomic DNA from the RNA (1 *µ*g) preparations was conducted by using DNase I, RNase-free (Thermo Fisher Scientific Inc., Waltham, MA, USA). The prepared RNA was reverse transcribed into cDNA according to the iScript™ Reverse Transcription Supermix for RT-qPCR (Bio-Rad Laboratories, Inc., Hercules, CA, USA). The synthesized cDNA was stored at −80°C. A reverse transcription-quantitative polymerase chain reaction (RT-qPCR) determined the messenger RNA (mRNA) levels for CYP1A2, GSTA1, GSTM1, and B2m genes (as an internal control) in each sample by using the PrimePCR™ SYBR® Green Assay of each gene (Cat no. 10025636, Bio-Rad Laboratories, Inc., Hercules, CA, USA). RT-qPCR was conducted with an iTaq™ Universal SYBR® Green Supermix (Bio-Rad Laboratories, Inc., Hercules, CA, USA) on a CFX96 Touch Deep Well RT-PCR System (Bio-Rad Laboratories, Inc., Hercules, CA, USA). The reaction conditions were as follows: polymerase activation and DNA denaturation at 95°C for 30 s, a total of forty cycles of denaturation at 95°C for 5 s, and annealing/extension at 60°C for 30 s. The 2^−ΔΔCt^ method was used to calculate the relative mRNA level of each gene [27].

### 2.8. Statistical Analysis

Data were reported as mean ± SD. The results were analyzed by using one-way ANOVA, followed by Bonferroni's post hoc test (multiple comparisons) using GraphPad Prism 5. A value of *P* < 0.05 was considered a statistically significant difference.

## 3. Results

### 3.1. Identification and Quantification of Fatty Acids

The fatty acid profile of the CO extracted from the fat of *Crocodylus siamensis* is depicted in Table 2. It was shown that the three fatty acids with the highest content were oleic (41.07%), linoleic (21.08%), and palmitic (19.92%) acids. Moreover, the omega-3 fatty acids were found at 1.18%, including alpha-linolenic acid (ALA) and docosahexaenoic acid (DHA) (Table 2).

### 3.2. CO-Induced Alteration in the Activities of CYP1A2 and GST

**Figure 1:**
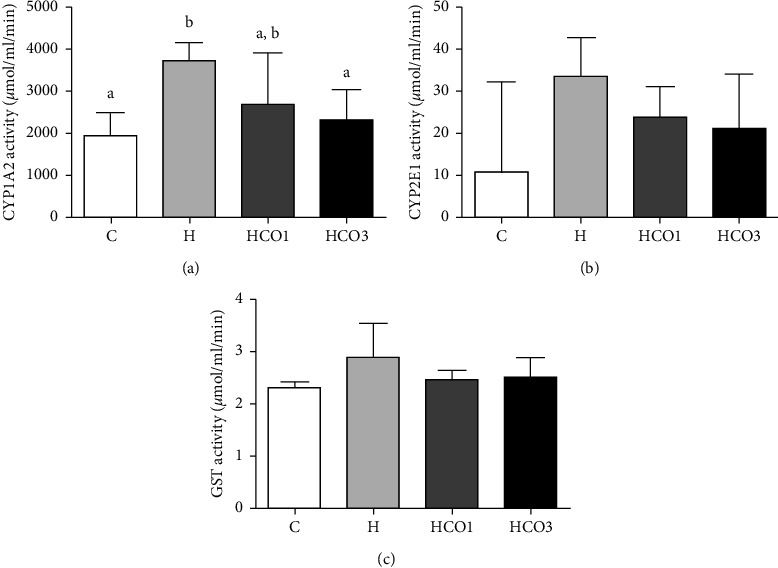
Plots of the enzyme activities of CYP1A2 (a), CYP2E1 (b), and GST (c) in the liver tissues of rats that were fed standard diet (C), HFD (H), and HFD with 1 ml (HCO1) and 3 ml (HCO3) per kg body weight of crocodile oil (*n* = 7/group). Different letters in the same graph show statistically significant differences between groups (*P* < 0.05).

The enzyme activity plots are shown in Figure 1. It was shown that HFD significantly increased the activity of CYP1A2 in the H group (3,720.47 ± 428.85 *µ*mol/ml/min). In contrast, their combined administration with 1 ml and 3 ml per kg body weight of CO normalized the activity of the enzyme and significantly reduced (*P* < 0.05) the activity of HCO3 group (2,303.73 ± 731.20 *µ*mol/ml/min) to their normal levels compared to the control group (1,941.00 ± 541.19 *µ*mol/ml/min) (Figure 1(a)). However, no significant difference was found in the activities of CYP2E1 and GST (Figures 1(b) and 1(c)).

### 3.3. Relative mRNA Expressions of CYP1A2, GSTA1, and GSTM1

**Figure 2:**
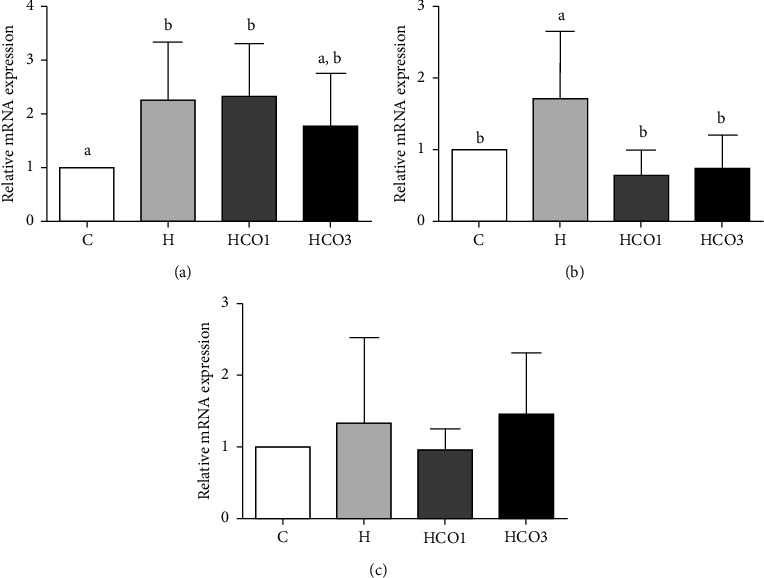
CYP1A2 (a), GSTA1 (b), and GSTM1 (c) messenger RNA (mRNA) expressions in the liver tissues of rats that were fed standard diet (C), HFD (H), and HFD with 1 ml (HCO1) and 3 ml (HCO3) per kg body weight of crocodile oil (*n* = 7/group). Quantitative reverse transcription polymerase chain reaction analysis was used to examine those mRNA levels. The data are presented as relative mRNA expression. Different letters in the same graph show statistically significant differences between groups (*P* < 0.05).

Quantitative RT-PCR determined the mRNA expressions of all samples to investigate the detoxification enzyme-related effects of CO supplementation on CYP1A2, GSTA1, and GSTM1 expressions in rats, as shown in Figure 2. The results showed that 3 ml per kg body weight of CO decreased the mRNA expression of CYP1A2 (1.758 ± 0.999) in the liver tissues of HFD-fed rats compared to the H group (2.250 ± 1.092) (Figure 2(a)). Moreover, 1 ml (0.640 ± 0.354) and 3 ml (0.744 ± 0.462) per kg body weight of CO significantly decreased (*P* < 0.05) the mRNA expressions of GSTA1 to similar levels to the control group (1.000 ± 0.000) when compared to the H group (1.712 ± 0.946) (Figure 2(b)). However, no significant difference was found in the expressions of GSTM1 (Figure 2(c)).

## 4. Discussion

This study aimed to investigate the effects of CO on the liver detoxification enzymes in rats by the oral administration of 1 and 3 ml per kg body weight of CO according to our previous studies [28, 29]. Since 1 and 3 ml per kg body weight of CO was suggested to benefit liver function by increasing energy metabolites, including oxaloacetate, and 3 ml per kg body weight of CO also ameliorated hepatic steatosis of the rats, the doses of CO were selected to be used in this study. The results of food consumption of the rats showed that the rats that received HFD had lower food intakes per day than those in the C group. However, due to the higher calories of the diet and the oil, groups of rats that received HFD had higher calories intakes per day (63.60–66.83 kcal/day/rat) compared to those in the C group (57.50 kcal/day/rat). Also, the percentages of body weight gain of the rats in H, HCO1, and HCO3 groups (9.50%, 8.47%, and 8.84%, respectively) were significantly higher (*P* < 0.05) than those in the C group (4.35%), suggesting that the obesity occurred in the HFD-fed groups (data not shown). Moreover, the serum lipid profile of the rats, including total cholesterol (CHOL), triacylglycerol, high-density lipoprotein cholesterol (HDL-C), and low-density lipoprotein cholesterol (LDL-C), was also investigated. However, significant difference was found only in the CHOL levels, of which the levels in HCO3 group significantly decreased compared to those in the C group (data not shown). Therefore, it could be suggested that oral administration of CO had no effect on serum lipid profile of the rats except for lowering the CHOL level compared to the C group.

This study revealed the increases in CYP1A2 activity and the mRNA expression in the HFD-fed group although other studies found opposite effects on various kinds of CYP450 [30–32]. Interestingly, the previous studies reported that HFD activated the aryl hydrocarbon receptor (AHR), which regulates lipid metabolism, vascular homeostasis, and metabolic dysfunction [33, 34]. Also, the increases in CYP1A1 and CYP1A2 expressions responded to the AHR agonists and AHR-dependent pathway as demonstrated in mice, rat, and human hepatocytes [17, 35, 36], although there is the dose-response divergence between the expressions of these two enzymes [37, 38]. Consequently, the increases in CYP1A2 activity and mRNA expression of the HFD-fed rats in this study might be attributed to the activation of AHR, which was affected by HFD ingestion.

However, the previous studies found that inhibiting CYP450 by essential oils constituents decreased formation of toxic metabolites, as CYP450 plays a vital role in procarcinogen activation [39, 40]. For instance, a study demonstrated the production of reactive oxygen species (ROS) stimulated by the overexpression of recombinant CYP1A1 and CYP1A2 in human lymphoblast-derived microsomes [41]. Also, the commercial fish oil decreased oxidative stress in the study of HFD-fed mice supplemented with fish oil [42]. Although a previous study found that long-chain unsaturated fatty acids, including oleic acid (approximately 40% in CO in this study), inhibit the activities of several CYP450s, including CYP1A2 [43], the results in this study suggested that the combination of fatty acids might help in attenuating this effect. Moreover, ALA is an omega-3 essential fatty acid that humans and other animals inevitably consume in their diet [44]. ALA can be converted into bioactive eicosapentaenoic acid (EPA) and then into DHA. Interestingly, the oil yields from this study presented ALA (0.96%) at similar levels compared with fish oil (1.45%) which is considered an omega-3-rich oil [45, 46]. Furthermore, it was demonstrated that administration of 2,3,7,8-tetrachlorodibenzo-p-dioxin (TCDD), which is an obesogenic-related ligand of the AHR, in omega-3 fatty acids-fed mice reduced the growth rates of lung carcinoma-derived tumors and inhibited their metastasis to lung and liver [47, 48]. This could suggest that omega-3 fatty acids could attenuate AHR activation and lead to other beneficial health effects. Since 3 ml per kg body weight of CO significantly normalized the activity and relative mRNA expression of CYP1A2 in the HFD-fed group to their normal levels (Figures 1(a) and 2(a)), the mechanism of liver protection could be suggested from the inhibition of the HFD-induced protein expression of CYP1A2 mediated by the omega-3 fatty acids found in CO.

Surprisingly, this study found significant decreases in the mRNA expressions of GSTA1 in the HCO1 and HCO3 groups compared to the H group. GSTs are multifunctional enzymes that not only catalyze the conjugation of electrophilic substrates to GSH but also conduct a range of other functions, including attenuating lipid peroxidation [15]. GSTA (alpha class GST) group, particularly GSTA1 and GSTA2, can catalyze GSH-dependent reduction of lipid hydroperoxides generated during oxidative stress [49, 50]. The GSTA also plays an essential cytoprotective role in detoxifying reactive electrophiles and products of lipid peroxidation [51, 52]. Moreover, a previous study reported that GSTA1 overexpression occurred to attenuate hydrogen peroxide-induced oxidative stress and protect the cells from responding to the associated cytotoxicity by attenuating lipid peroxidation [53]. Accordingly, it could be suggested that the increased GSTA1 mRNA expression in the H group might be attributed to higher lipid peroxidation occurred by the HFD consumption. However, CO reduced the effects of HFD, which led to the reduction of lipid peroxidation related to GSTA1 expression.

In contrast, the mRNA expressions of GSTM1 in this study had no significant difference among the groups. The GSTM (mu class GST) functions in detoxifying electrophilic compounds, including carcinogens, therapeutic drugs, environmental toxins, and products of oxidative stress, by conjugating with GSH [54]. High GSTM expression is important in preventing chemical mutagens and carcinogens [54]. Nevertheless, it was found in relatively low amounts in the liver and the other organs [55, 56]. Consequently, it could be suggested that HFD and CO might not be contributed to the formation of carcinogens and other xenobiotics, which activate the expression of GSTM1.

Furthermore, in contrast to the result, HFD-fed rats were found to have decreased specific activities of hepatic antioxidant enzymes, including CAT, glutathione peroxidase (GPx), glutathione reductase (GR), and GST, and decreased GSH levels that were reversed into increased activities by *Capparis spinosa* fruit extract treatment in a dose-dependent manner [57]. Also, it was indicated that HFD induced accumulation of ROS and the downregulation of the enzyme-related gene expressions that control the neutralization of such oxidative stress [58, 59]. However, in this study, the H group's relative mRNA expression of GSTA1 was significantly higher than the HCO1 and HCO3 groups. Therefore, this could indicate why the GST activity in the H group was slightly higher than that in the others. However, there were no significant differences in the GST activities among the groups.

## 5. Conclusions

The enzymatic and molecular effects observed in the liver tissue, together with the fatty acid composition of CO, may contribute to the hepatoprotective effects presented in the HFD-fed animals treated with the oil. The results provided evidence that the use of CO could mainly help in the liver detoxification mechanism through decreases in CYP1A2 activities and mRNA expression even when consumed with an obesogenic diet. Further investigation on the correlation between CO and the inhibition of HFD-induced AHR-dependent pathway should be performed to ensure the liver protective effects induced by CO.

## Figures and Tables

**Table 1:** Formulations of standard diet and high-fat diet (HFD).

Diet components	Standard diet (g/100 g diet)	HFD (g/100 gdiet)
Moisture	8.90	N/D
Crude protein	24.90	20.18
Fat	4.60	41.04
Fiber	4.10	5.35
Nitrogen-free extract (NFE)	51.00	27.46
Crude ash		
Calcium	1.06	1.25
Phosphorus	0.99	0.97
Sodium	0.31	0.12
Chlorine	N/D	0.18
Others	4.24	4.74

**Table 2:** The fatty acid profile of crocodile oil obtained from *Crocodylus siamensis* (data are expressed as mean ± standard deviations of triplicate measurements).

Fatty acid	% methylated content
Lauric acid	0.11 ± 0.036
Myristic acid	0.57 ± 0.060
Palmitic acid	19.92 ± 0.307
Stearic acid	5.42 ± 0.029
Arachidic acid	0.13 ± 0.012
Myristoleic acid	0.10 ± 0.006
Palmitoleic acid	3.83 ± 0.137
Heptadecenoic acid	0.11 ± 0.010
Oleic acid	41.07 ± 0.549
Linoleic acid	21.08 ± 0.180
Alpha-linolenic acid (ALA)	0.96 ± 0.049
Gamma-linolenic acid	0.18 ± 0.006
Eicosenoic acid	0.41 ± 0.026
Eicosadienoic acid	0.27 ± 0.035
Eicosatrienoic acid	0.27 ± 0.035
Arachidonic acid	0.82 ± 0.092
Docosahexaenoic acid (DHA)	0.22 ± 0.015

## Data Availability

The data that support the findings of this study are available from the corresponding author upon reasonable request.
